# Morphological differentiation and its application in industrial filamentous fungi

**DOI:** 10.1080/21501203.2026.2646436

**Published:** 2026-04-15

**Authors:** Yushi Fu, Yuanyuan Pan, Yongpeng Yao, Gang Liu

**Affiliations:** a State Key Laboratory of Microbial Diversity and Innovative Utilization, Institute of Microbiology, Chinese Academy of Sciences, Beijing, China; b College of Life Sciences, University of Chinese Academy of Sciences, Beijing, China

**Keywords:** Filamentous fungi, morphogenesis, polarized growth, hyphal branching, cell envelope homeostasis, morphology engineering, machine learning

## Abstract

Filamentous fungi are essential ecosystem members and major platforms for industrial biomanufacturing, where their morphological differentiation strongly influences broth rheology, mass/oxygen transfer, secretion efficiency, stress tolerance, and ultimately productivity. However, the intricate network in which process-specific cues are sensed and integrated to reprogram the endogenous developmental pathways remains incompletely understood, limiting rational morphology engineering of the industrial filamentous fungi. This review summarizes recent advances in the regulation of morphological differentiation of filamentous fungi, integrating internal and external signaling along with molecular control of spore germination, polarity establishment, hyphal extension/branching, and cell wall-plasma membrane homeostasis. We discuss how distinct morphologies affect performance in submerged and solid-state fermentation, and we overview the practical control strategies including inoculation design, environmental and hydrodynamic optimization, microparticle-enhanced cultivation, and genetic reprogramming. Finally, we highlight emerging monitoring approaches and recent progress in machine learning and modeling within AI-enabled biomanufacturing, proposing an integrated gene–morphology–function–titer perspective for next-generation fungal cell factories.

## GRAPHICAL ABSTRACT

**Figure:**
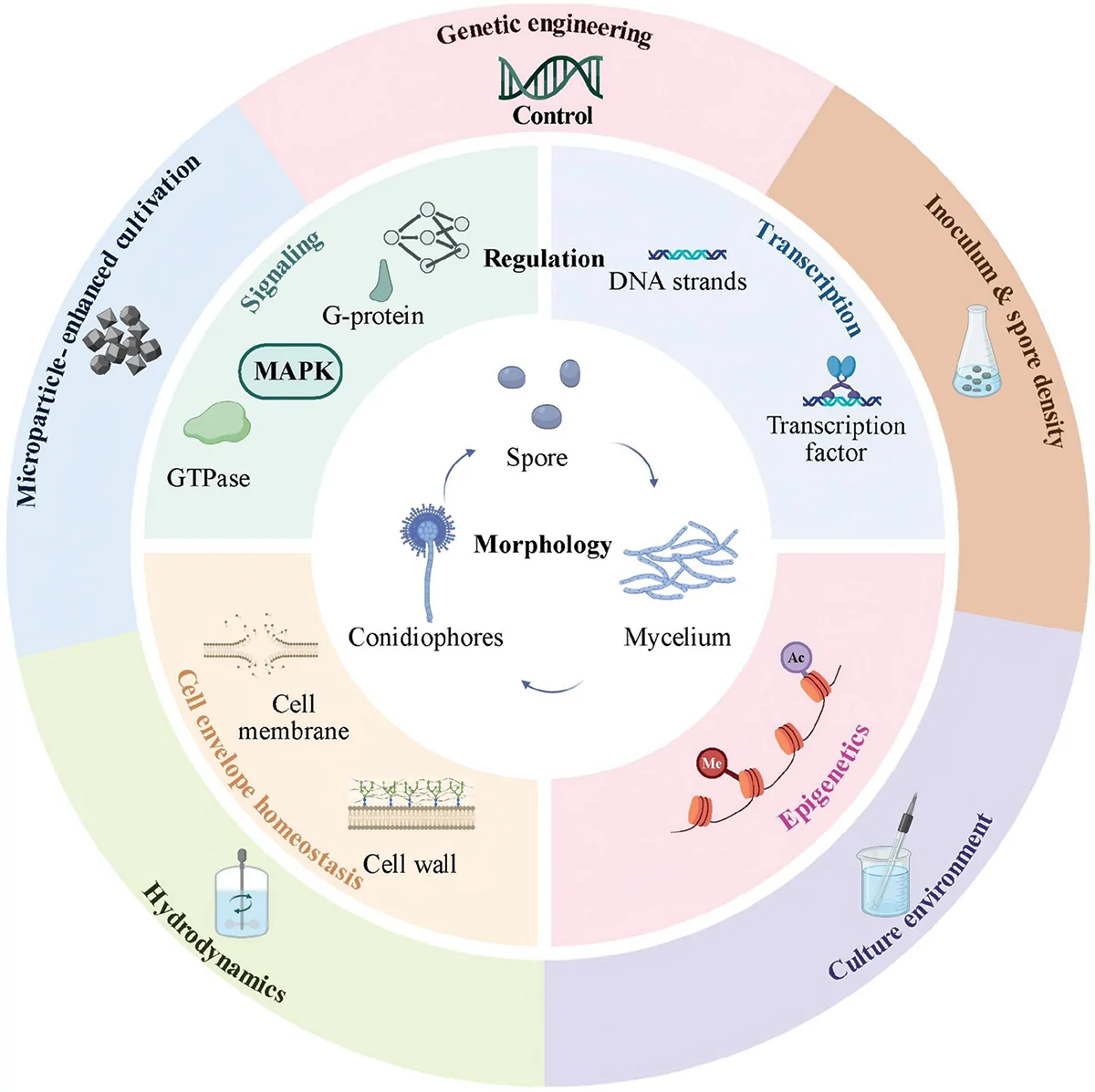

## 1. Introduction

Filamentous fungi play indispensable roles in natural ecosystems, industrial and agricultural production. On the one hand, they are key contributors to maintaining ecosystem stability due to their functional diversity (Zheng et al. 2020). On the other hand, they exhibit great potential for exploitation in industrial processes (Peng et al. 2021) and agricultural applications (Tyśkiewicz et al. 2022). Of them, *Trichoderma reesei*, *Aspergillus niger*, and *Penicillium chrysogenum* are the outstanding representatives of the industrial filamentous fungi for producing cellulases, organic acids, and penicillin, respectively.

Filamentous fungi typically undergo a complete morphological differentiation cycle which starts from spore germination and is followed by mycelial formation and large-scale synthesis of metabolic products. Finally, the reproductive structures are formed under stress or developmental cues. At each stage, the morphogenesis is strongly related to biosynthesis and secretion of primary and secondary metabolites. Thus, there is a tight correlation between the morphological differentiation of filamentous fungi and their industrial performance.

Morphological differentiation of filamentous fungi is a highly programmed process. Spore germination, as the starting point of their life cycle, is accompanied by complex biochemical changes and establishment of polarity (Martin-Vicente et al. 2020), in which the Ras signaling network plays a conserved and central regulatory role (Fortwendel 2015). During vegetative growth, branch formation is governed by finely tuned spatial regulation and morphogenetic mechanisms (Harris 2019). Hyphal fusion, which depends on the MAPK cascades and related pathways, builds an intricated three-dimensional network that is essential for efficient distribution of resources, signaling, and colony morphogenesis (Fleißner and Herzog 2016). These mechanisms are fundamental to our understanding of fungal development and provide a theoretical basis for improving strain performance through morphological engineering (Lu et al. 2024).

Under industrial conditions, the presence of high shear forces, complex substrates, and non-steady-state operating regimes causes generation of numerous process-specific signals and stresses. There remains a deficiency in systematic studies regarding the perception and integration of these signals by fungal cells, as well as how these cells subsequently reprogram their intrinsic developmental pathways for industrial productivity. This gap impedes our capacity to rationally reprogram the morphogenesis of industrial strains to more effectively align with specific process requirements.

In this review, the delineation of the typical life cycle and morphogenesis of filamentous fungi, the internal and external signaling networks and molecular mechanisms that govern morphological differentiation, the evaluation of morphological structures influencing production performance within various fermentation processes, and the intrinsic connections between morphology and industrial productivity are summarized. Additionally, the current strategies for morphological control, the challenges, and future directions in this field are discussed and predicted. Some excellent previous reviews on the morphological differentiation of filamentous fungi are referred to in the following text.

Relevant literature was retrieved from PubMed and Web of Science (past ~10 years, with selective inclusion of seminal earlier studies) using keyword combinations covering filamentous fungi, morphology/morphogenesis, production traits (organic acids, enzymes, secondary metabolites), bioprocess conditions, and modeling (image analysis, high-throughput quantification, machine learning/deep learning). English-language original studies and key reviews linking morphology to process and/or production were included, while clinically focused studies without process- or production-relevant morphology–product relationships were excluded.

## 2. Morphological differentiation in filamentous fungi

### 2.1. Spore germination of filamentous fungi

Spore germination signifies the transition from dormancy to metabolically active growth and is a key developmental event in the life cycle of filamentous fungi. It typically proceeds through three stages including dormancy breaking, isotropic swelling, and polarized growth. During dormancy breaking, the fungal spore switches from anaerobic to aerobic respiration by using stored compounds for energy (Osherov 2001; Novodvorska et al. 2016). In the stage of isotropic swelling, the fungal spore takes up water and undergoes pronounced volume expansion, accompanied by cell wall remodeling and altered mechanical properties. Finally, the fungal spore forms a germ tube and elongates into a hypha, completing the transition from spore to mycelium by polarized growth. These stages are interdependent, and perturbation at any step can delay or abort fungal spore germination.

Fungal spore germination is tightly regulated by the environmental cues such as temperature, nutrient availability, and exogenous signals (Bärlocher et al. 2013). Nutrients not only supply essential materials and energy for fungal growth but also often act as regulatory signals. In *Neurospora crassa*, both defined (BM) and natural (MSM) media affect spore germination, hyphal growth, and the expression of metabolism-related genes (Wang et al. 2019). In *A. niger*, spores exhibit a stringent structural selectivity for nitrogen sources, where only the specific L-amino acid-related compounds can trigger germination (Hayer et al. 2014). Similarly, carbon sources can also act as initiating signals. Trehalose degradation and isotropic swelling are induced after spores specifically sense the D-glucose and its related sugars such as D-mannose and D-xylose in *A. niger* (Hayer et al. 2013). There is also a close coupling between the sensing of exogenous carbon and the mobilization of internal reserves. Lipid metabolism plays a significant role in *Aspergillus fumigatus*, where lipoxygenase activity enhances the programmed germination, especially in environments containing polyunsaturated fatty acids (Fischer et al. 2017). This suggests that these lipids and their oxidation products may contribute to the modulation of membrane properties and lipid-mediated signaling during fungal spore germination.

As one of the major exogenous signals, light influences fungal development through photoreceptor-mediated signaling pathways. Activation of the red-light photoreceptor FphA is associated with the increased HogA phosphorylation and its nuclear accumulation in *Aspergillus alternata*, suggesting an interaction of light with the high-osmolarity glycerol (HOG) MAPK pathway (Igbalajobi et al. 2019). Exactly the same, the bZIP transcription factor PcAtfA responds to light and regulates spore germination in *P. chrysogenum* (Wolfers et al. 2015).

Fungal spore germination is regulated by multiple interconnected signaling pathways. Reactive oxygen species (ROS) and nitric oxide (NO) have been identified as important signaling molecules in spore germination in several fungi. In *Puccinia striiformis*, scavenging NO or ROS markedly delays spore germination and inhibits the elongation of the germ tube, whereas moderate supplementation of NO or ROS restores its normal development (Yin et al. 2016). Confocal imaging demonstrates that NO and ROS are predominantly accumulated at the germ pore and the growing hyphal tip in an appropriate ratio, which coincides with fungal polarized growth. The Ras-GTPase pathway is also essential for fungal germination and polarity. In several filamentous fungi, deletion of the Ras-GTPase encoding gene *rsrA* results in their reduced growth and delayed germination, suggesting RsrA is required for fungal polarized growth (Cano-Domínguez et al. 2023). However, overexpression of *rsrA* leads to severe morphological defects and the formation of multiple growth axes (Martin-Vicente et al. 2020), indicating the activity of RsrA is tightly regulated during fungal development.

Epigenetic regulation—particularly histone modifications—may contribute to the transition of spores from dormancy to germination. Single-cell transcriptomic analysis of fission yeast spores revealed dynamic changes in histone H3-related chromatin features/marks associated with early germination (Tsuyuzaki et al. 2020). In *Aspergillus flavus*, the H3K4 histone methyltransferase Set1 is required for normal spore germination, hyphal development, and mycotoxin biosynthesis (Liu et al. 2020).

In conclusion, spore germination is influenced by environmental factors such as nutrients, temperature, and light, which interact with intracellular signaling pathways, including ROS/NO and Ras-GTPase, as well as chromatin regulation mechanisms involved by the enzymes like Set1. From a practical perspective, the most effective interventions for manipulating process-control variables, such as medium composition and feeding strategy, temperature, and water activity, and light programs, are selected based on their scalability, non-genetic nature, and ease of optimization to enhance synchrony and early development. Conversely, the spatial dynamics of ROS/NO and signaling linked to lipid oxidation are primarily of theoretical interest, as the limited understanding of their sensors, pathway hierarchy, and cross-species applicability continues to impede the development of predictable interventions.

### 2.2. Hyphal extension and its regulation in filamentous fungi

Hyphal extension and branching are fundamental to growth, colonization, and spatial expansion of filamentous fungi. They are primarily characterized by continuous, polarized tip growth, and involve a series of cytological events. This process depends on a highly active secretory system that delivers membrane, proteins and cell wall materials to the apex (Roberson 2020). The Spitzenkörper (Spk), a structure that is rich in vesicles, cytoskeletal elements, and signaling proteins, is located at the hyphal tip and functions as an organizing center for vesicle trafficking, cell wall deposition, and growth directionality (Figure 1). Subsequently, new polarity sites arise at the apical or subapical regions to produce apical or lateral branches, and branching fundamentally involves re-establishment of polarity axes.

**Figure 1.**
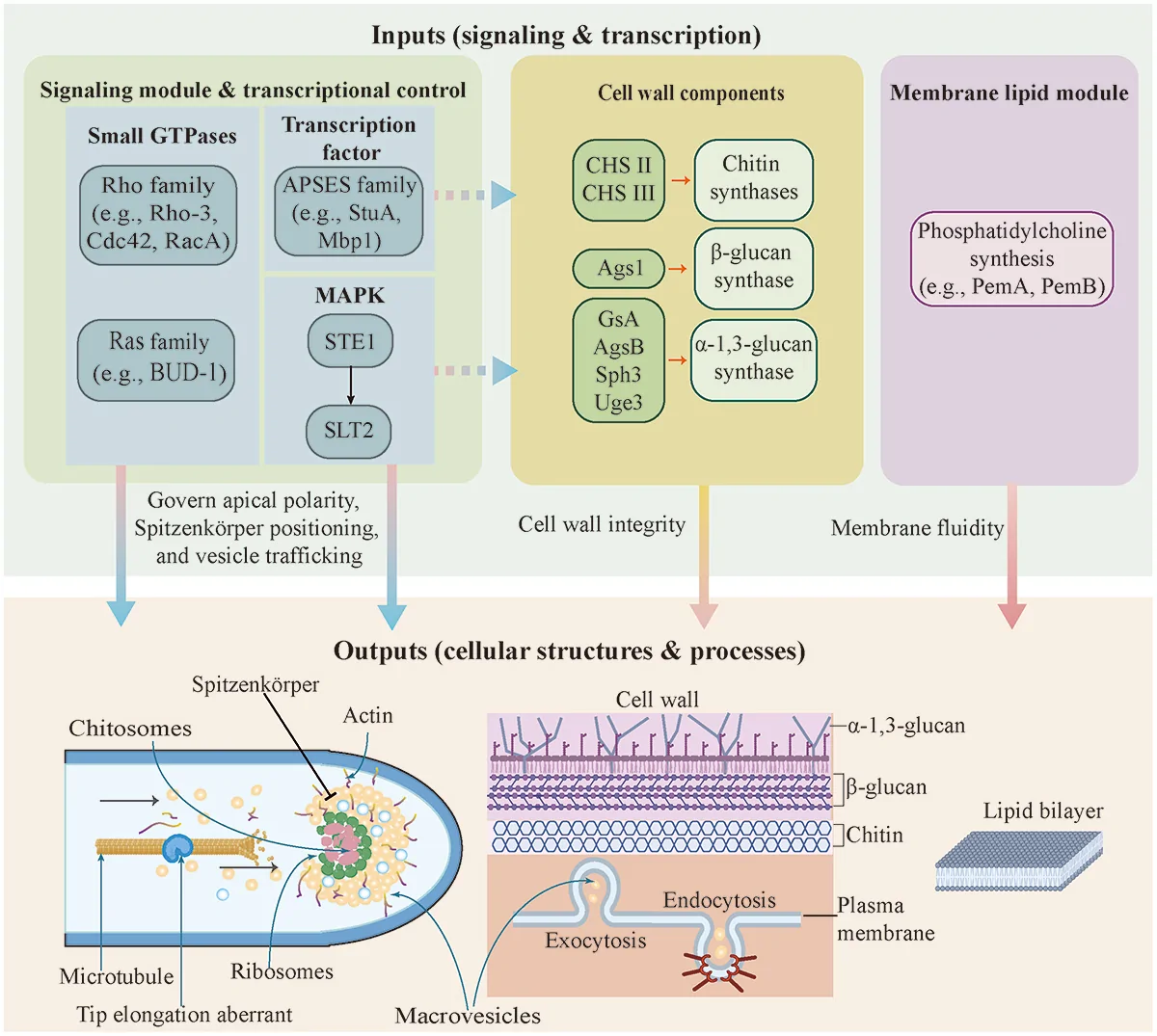
Inputs and outputs underlying polarized hyphal tip growth and branching in filamentous fungi. Schematic summary of inputs and outputs underlying hyphal polarity establishment and branching in filamentous fungi, as discussed in this review. The inputs include small GTPases, MAPK cascade, and APSES-family transcription factors, which collectively govern apical polarity, Spitzenkörper positioning, and vesicle trafficking. Cell-wall and membrane biogenesis modules include chitin synthases, β-glucan synthesis, α-1,3-glucan synthesis, and proteins involved in phosphatidylcholine (PC) synthesis, thereby shaping cell-wall integrity and membrane properties. Outputs include cytoskeleton-guided transport and vesicle accumulation at the Spitzenkörper, exocytosis-driven delivery and deposition of wall materials at the plasma membrane, and endocytosis-mediated recycling to maintain membrane homeostasis. Together, coordinated regulation of cell-wall assembly and membrane dynamics supports sustained tip growth and hyphal morphogenesis. Dashed arrows indicate indirect regulatory relationships. MAPK, mitogen-activated protein kinase.

Polarized growth is dependent on the dynamic remodeling of the cytoskeleton, wherein secretory vesicles are transported along cytoskeletal tracks and subsequently fuse at the apex to facilitate membrane insertion and cell wall expansion (Berepiki et al. 2011; Callejas-Negrete et al. 2015; Riquelme et al. 2018). Tip elongation aberrant (Tea) proteins function as crucial “end markers” that collaborate with the cytoskeletal regulators to direct vesicle targeting and establish new polarity axes. The absence of Tea results in a loss of directional growth, as well as diminished septation and branching (Arellano et al. 2002; Qu et al. 2022; Rogers et al. 2024). Concurrently, endocytosis plays a role in recycling excess plasma membrane and essential membrane proteins, thereby modulating apical membrane composition and tension. Both clathrin-mediated and AP-2 dependent clathrin-independent pathways are involved in the selective uptake of cargo, influencing the spatial distribution of apical membrane proteins and lipids (Bedsole et al. 2025). The composition of membrane lipids serves as an important determinant of hyphal morphogenesis. For example, modifications in phosphatidylcholine (PC) synthesis have a profound impact on the hyphal extension and differentiation of *Aspergillus oryzae* (Suzawa et al. 2024).

At the signaling level, protein kinases, small GTPases, and transcription factors constitute the major regulatory networks that regulate hyphal extension and branching (Figure 1). In *Monascus* spp., the serine/threonine kinase MpSTE1 directly influences branching and secondary metabolism through the phosphorylation of targets involved in the cell cycle, cytoskeleton organization, and signaling (Duan et al. 2024). Ras-like GTPases and Rho-like GTPases play a key role as molecular switches for polarity and tip-directed secretion. The Ras-like GTPase BUD-1 localizes to the growing tips during spore germination and hyphal growth and its deletion severely impairs fungal growth rate and directionality (Cano-Domínguez et al. 2023). RHO-3 colocalizes with glucan synthase (GS-1) and chitin synthase (CHS-1) in the macrovesicle zone of the Spitzenkörper, and its absence reduces aerial hyphal length, supporting the idea that RHO-3 contributes to the apical targeting during cell wall synthesis and polarized growth (Ornelas-Llamas et al. 2024). The transcription factors associated with cell wall formation significantly influence hyphal extension. For instance, the APSES transcription factor MBP1 is required for normal growth, as well as for the synthesis of β-glucan and chitin. Deletion of *mbp1* reduces fungal growth and causes notable morphological defects (Kojima et al. 2024), highlighting the close link between cell wall formation and tip growth. It is similar in the cephalosporin C (CPC) producing strain *Acremonium chrusogenum*, an APSES transcription regulator AcstuA has been found to be involved in conidiation, cephalosporin biosynthesis, and cell wall integrity (Hu et al. 2015).

Overall, several modules are already actionable for strain improvement and process optimization, particularly those that control biomass and morphology, such as cell wall biosynthesis, membrane lipid metabolism, and secretory capacity. These can be tuned via genetic engineering, and in some cases through process control (e.g. nutrient and environmental conditions) that shift membrane and wall demands during tip growth. By contrast, the spatiotemporal organization of the Spitzenkörper, end-marker polarity circuits, and the mechanistic coupling between endocytosis, membrane tension, and growth directionality remain primarily of fundamental interest and are currently less tractable for predictable intervention.

### 2.3. Conidiation of filamentous fungi

The formation of spores is crucial for the survival and propagation of filamentous fungi. Conidia are produced in large quantities within a short period for rapid dispersal and colonization. The majority of *Aspergillus* and *Penicillium* species produce conidia on conidiophores that are differentiated from vegetative hyphae. Initially, aerial hyphae undergo apical swelling to form a vesicle in which extensive nuclear division occurs. Subsequently, phialides emerge from the vesicle surface, incorporate migrating nuclei, and finally generate chains of conidia through repeated asymmetric divisions. In *Aspergillus*, asexual development is regulated by upstream developmental activators (uDAs) and the central developmental pathway (CDP, BrlA–AbaA–WetA). uDAs initiate the transition from vegetative growth to conidiophore development and activate the CDP, while the CDP regulators interact to drive conidiophore formation and conidial maturation (Garzia et al. 2010; Wu et al. 2018). This “uDAs–CDP” module has been considered as the classical model for studying *Aspergillus* asexual development (Figure 2).

**Figure 2.**
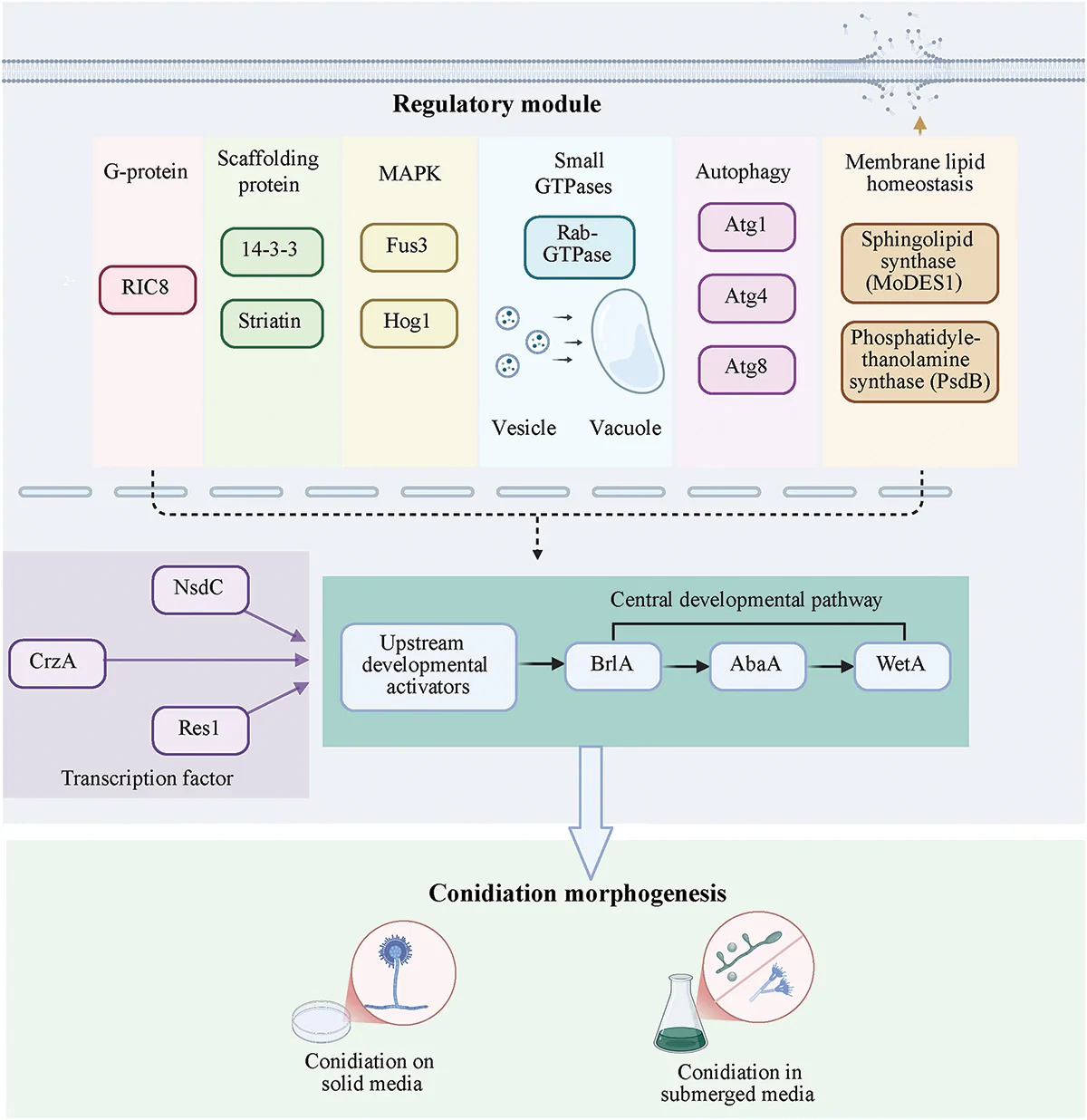
Overview of representative regulatory modules implicated in conidiation of filamentous fungi. Schematic overview of the selected regulatory modules controlling conidiation in filamentous fungi under different culture conditions, as discussed in this review. Upstream inputs include a G-protein signaling module, MAPK pathways, and scaffold proteins within these pathways. Rab-family small GTPases link vesicle trafficking and vacuolar dynamics to developmental outputs. Autophagy components and membrane lipid homeostasis factors contribute to cellular remodeling during morphological differentiation. Transcriptional factors further drive upstream developmental regulators to initiate the conserved central cascade BrlA-AbaA-WetA, ultimately triggering conidiation. The lower panel depicts conidiation on solid media and in submerged culture. Solid arrows indicate the regulatory direction, whereas dashed arrows denote the putative indirect or multistep connections. MAPK, mitogen-activated protein kinase.

Various signaling and transcriptional modules have been demonstrated to influence conidiation, morphology, and spore maturation of filamentous fungi. Branches of the mitogen-activated protein kinase (MAPK) pathways exert species-specific and pathway-specific effects on conidiation. Deletion of *mpkB*, the ortholog of *Saccharomyces cerevisiae* FUS3, significantly reduces conidiation and leads to conidial abnormalities in *A. flavus* (Jun et al. 2020). Conversely, loss of MaSln1, which is an upstream membrane protein in the SLN1 branch of the high-osmolarity glycerol (HOG)-MAPK pathway, changes the conidiation pattern without affecting the total spore output in *Metarhizium robertsii* (Wen et al. 2022). In *N. crassa*, the deletion of the Gα subunit RIC8 induces conidiation in the submerged culture, potentially by altering the expression of genes encoding vacuolar amino acid transporters, and in turn perturbing the compartmentalization of arginine and ornithine between the cytosol and the vacuole (Quinn et al. 2024).

Small GTPases regulate conidiation by managing vesicle trafficking and endomembrane functions (Tang et al. 2023). In *Arthrobotrys oligospora*, the Rab-GTPase AoRab7A influences trap formation and conidiation by affecting vacuole assembly and protein degradation (Zhu et al. 2024). In addition, the scaffold proteins like 14-3-3 protein ArtA of *A. flavus* and striatin-like protein FvSTR1 of *Fusarium virguliforme* are crucial for linking asexual development with secondary metabolism, and coordinating conidiation with pathogenicity (Islam et al. 2017; Ibarra et al. 2018).

The regulatory network consists of a diverse of transcription factors strictly controls fungal conidiation. In *A. fumigatus*, deletion of the transcription factor NsdC results in abolition of sexual reproduction, reduction in conidiation on solid media, but a notable increase of conidiation in submerged culture (Alves de Castro et al. 2021). Interestingly, the CCAAT-binding complex (CBC) has been identified as a negative regulator of conidiation in *A. fumigatus*. Loss of CBC leads to elevation of the intracellular Ca^2+^ level, which activates the calmodulin-dependent transcription factor CrzA, thereby enhancing conidiation of *A. fumigatus* in liquid culture (Ren et al. 2021). In *Thermothelomyces thermophilus*, deletion of the Zn(II)_2_Cys_6_ transcription factor Res1 completely abolishes the fungal conidiation in submerged culture, while conidiation on solid media remains unaffected (Drescher et al. 2024). Together, these studies highlight condition-dependent regulation of asexual development, where the same regulator can exert distinct effects in submerged versus solid cultures.

Cellular architecture and membrane lipid homeostasis are essential for normal conidiation processes. In *Magnaporthe oryzae*, deletion of the sphingolipid synthase gene *MoDES1* disrupts lipid homeostasis, cell wall integrity (CWI), and the Osm1-MAPK signaling pathways, and in turn inhibits conidiation (Wang et al. 2024). Similarly, the biosynthesis of phosphatidylethanolamine (PE) relies on the enzyme phosphatidylserine decarboxylase PsdB in *Aspergillus nidulans*. Mutants lacking *psdB* exhibit significantly reduced conidiation and abnormal morphogenesis (Takagi et al. 2021), implying the importance of membrane lipid composition in asexual development. Furthermore, the autophagy-related genes have been demonstrated to play critical regulatory roles in asexual spore formation (Voigt and Pöggeler 2013; Corral-Ramos et al. 2015). For example, disruption of the serine/threonine kinase gene *Acatg1* clearly inhibits the autophagosome formation under starvation and results in reduction of conidiation in *A. chrysogenum*, suggesting that intracellular recycling and stress tolerance are intricately linked to spore development (Wang et al. 2014).

Overall, conidiation is controlled by the conserved uDAs–CDP program alongside multiple signal-transduction layers (Figure 2). Because conidiation is often coupled to secondary metabolite production and reduced yields of recombinant proteins, industrial processes frequently require tuning conidial output to match production goals. From strain engineering, the most readily actionable targets are core developmental regulators and transcription factors, which can be adjusted through targeted genetic modulation. By contrast, regulators embedded in broader signaling networks and lipid homeostasis are often pleiotropic and difficult to decouple from downstream effects, and therefore remain challenging to engineer directly and are still studied mainly in a fundamental context.

## 3. Fungal morphogenesis correlated with industrial productivity

Filamentous fungi are extensively utilized in producing industrial enzymes, organic acids, and high-value secondary metabolites. Numerous studies have demonstrated that the industrial productivity of filamentous fungi is significantly influenced by their morphological differentiation (Papagianni and Mattey 2006; Zhang et al. 2007). Some factors such as hyphal branching, pellet formation, flocculent aggregates, development of sclerotia or microsclerotia, as well as colonization on solid substrates, are not only correlated with mass transfer and rheological properties during fermentation but also have substantial effects on protein secretion, exoenzyme profiles, and secondary metabolite biosynthesis.

### 3.1. Impact of mycelial pellets and flocculent aggregates on industrial productivity

It has been found that mycelial pellets and flocculent aggregates are either positively or negatively related to the industrial productivity of filamentous fungi. These morphological structures are often associated with either improved product accumulation—potentially via better rheology and mass transport—or with diffusion limitations and undesirable morphological shifts. Thus, understanding the correlation between these structures and their metabolic responses is crucial for optimizing the fermentation process of industrial strains.

During organic acid production, compact pellet formation is generally advantageous as it reduces broth viscosity and enhances mixing, thereby facilitating high cell-density cultivation (Latif et al. 2025). Concurrently, the dense structure of these pellets causes the limited oxygen solubility in the hyphal bulb and the moderate energy constraints, such as the reduction of oxidative phosphorylation. Interestingly, such constraints in compact pellets promote citrate production, potentially by shifting carbon flux toward organic acid accumulation (Wang et al. 2015). Conversely, the dispersed and highly branched mycelial structures are typically favored for production of extracellular enzymes. Presumably, the hyphal tips, as the primary sites for protein secretion, are more exposed in such structures, which is beneficial to produce enzymes (Müller et al. 2002). Meanwhile, strains with highly branched mycelia have been reported to exhibit relatively stable carbohydrate flux, which may help meet the energy demand for both mycelial growth and protein production (Sakuragawa et al. 2021). However, extensive dispersion significantly increases viscosity and adversely affects rheology, necessitating higher agitation. In turn, this may result in shear damage, which is often associated with physiological instability and process-performance fluctuations in industrial strains (Walisko et al. 2012). Therefore, an optimal balance between adequate dispersion and acceptable viscosity is essential.

In detail, the size, density, and internal structure of pellets play a crucial role in determining diffusion and transport of substrates with products, even in oxygen penetration. Oxygen penetration in outer region of the pellet is closely associated with the local density of hyphae. High density of hyphae causes rapid oxygen depletion and formation of the distinct oxygen-limited zones in the pellet (Deffur et al. 2024). Similarly, the structure of the pellet significantly influences the transport of substrates and products. Substrates need to penetrate the outer layer to reach the metabolically active inner cells, and excessive resistance can impede the growth and metabolism of these inner hyphae (Zhu et al. 2023). Obstruction of product efflux may result in local accumulation and subsequent changes in pH or ionic conditions, which affect cellular activity and product stability. Consequently, the architecture of the pellet is a key contributor to the behavior of product diffusion and impacts the final industrial productivity (Lu et al. 2017). Consideration of mycelial pellets and flocculent aggregates is particularly critical for products sensitive to mass transfer, such as organic acids and certain secondary metabolites.

### 3.2. Effect of shear stress on fungal morphology and productivity

In stirred-tank fermentation, shear stress is a key parameter for industrial productivity of filamentous fungi. Shear stress affects fungal morphology, vesicle transport, and metabolite biosynthesis. Shear stress can mechanically alter mycelial aggregation and modify metabolic fluxes through its effects on secretion and oxygen transfer, thereby exerting a dual influence on industrial productivity.

In *A. niger*, variations in agitation affect the distribution of secretory vesicles and glucoamylase production (Kunz and King 2022). Excessive shear stress leads to a reduction in vesicle numbers and disrupts apical secretion, thereby suppressing extracellular enzyme production, while moderate shear stress maximizes glucoamylase production. This suggests the existence of an “optimal shear window,” wherein moderate shear prevents excessive aggregation and enhances mass transfer without compromising the secretion machinery.

At the process scale, shear is typically correlated with the volumetric oxygen transfer coefficient (*K*_*L*_*a*) and plays a critical role in the biosynthesis of secondary metabolites. In *Fusarium oxysporum*, insufficient shear and oxygen availability limit both growth and bikaverin production, while excessive shear damages hyphae and reduces the pigment formation. Optimal combinations of shear and *K*_*L*_*a* are necessary to improve bikaverin yields (dos Santos et al. 2022). Notably, the reported effects of shear stress are not always consistent across species or with product classes. This variability is frequently attributed to variations in dominant morphology, cell-wall robustness, and product physiology, which influence the balance between the enhanced dispersion/mass transfer and the shear-induced hyphal damage or impaired secretion. Furthermore, cross-study comparisons are often constrained by inconsistent definitions and estimation methods of shear stress (e.g. wall shear stress derived from computational fluid dynamics versus apparent shear stress based on rheology and correlation), as well as by the intrinsic coupling of shear with oxygen transfer and mixing. Therefore, in industrial scale-up, shear should be considered a modifiable signal for influencing morphology and industrial productivity of filamentous fungi.

### 3.3. Environment-triggered morphological differentiation and industrial productivity

Beyond aggregate structure and hydrodynamic forces, process-relevant chemical and nutritional cues can also reprogram developmental trajectories, generating differentiated morphotypes that are closely associated with productivity performance. During cultivation, *A. chrysogenum* produces two types of asexual spores including conidia and arthrospores. The formation of conidia is originated from the differentiation of hyphal branches in the solid medium. On the other hand, arthrospores are formed during liquid fermentation and referred to as “yeast-like” cells. Under nutrient depletion or upon methionine supplementation during fermentation, *A. chrysogenum* has been reported to form arthrospores. Concurrently, CPC begins to be produced in large quantities, suggesting a close correlation between arthrospore formation and CPC production. Nash and Huber (1971) found that the highest CPC production occurs when arthrospores or yeast-like cells are formed in *A. chrysogenum*. In addition, the hyphae of *A. chrysogenum* are generally thinner in sulfate containing media. However, the hyphae will expand and form arthrospores, and in turn, fragmentation increases after adding methionine, which is consistent with CPC production. Therefore, the formation of arthrospores is often used as a practical morphological proxy for CPC productivity in industrial settings. To reveal the mechanism, Long et al. (2012) used T-DNA random insertion mutagenesis to obtain a series of genes related to morphological differentiation of *A. chrysogenum*. Of them, disruption of *AcsepH* resulted in a 9-fold decrease in conidiation and the production of CPC also significantly decreased. Such associations between morphological differentiation and productivity have been reported across many filamentous fungi.

### 3.4. Effects of morphological differentiation on productivity in solid-state fermentation

In solid-state fermentation, the morphological differentiation plays a critical role in fungal colonization and expansion across the substrate surface, and it can substantially affect enzyme production efficiency and overall process stability. For instance, surface imaging of *Penicillium simplicissimum* demonstrated that the static versus intermittently mixed solid-state fermentation caused markedly different hyphal distribution and colonization patterns. These differences were accompanied by changes in surface expansion, penetration depth, and the characteristics of local microenvironments such as moisture and oxygen profiles (de Almeida et al. 2017). The morphological changes induced by these operational conditions may in turn influence fungal physiology and metabolic activity, thereby exerting an indirect yet essential regulation on bioproduct formation. Studies on glucoamylase production by *Rhizopus oryzae* in solid-state fermentation provide further evidence for an association between fungal morphology and industrial productivity. On solid media with suitable moisture and pore structure, the hyphae exhibit significant thickness, extensive branching, and uniform distribution, supporting a hyphal growth rate of *μ* = 0.15 h^−1^ and glucoamylase activity of 55 U/g (Tang et al. 2018). This mechanically stable 3D network maintains an active hyphal front and sustained secretion, ensuring high glucoamylase production and overall fermentation efficiency. Thus, morphological differentiation in solid-state fermentation may influence industrial productivity through at least two aspects. One is the fungal colonization patterns and hyphal growth rates shaped by process parameters such as cultivation mode, substrate moisture, and physical structure. Another is the traits including hyphal thickness, branching density, and spatial uniformity, dictate mechanical stability and sustained secretion.

## 4. Morphological control of industrial filamentous fungi

The morphology of industrial filamentous fungi, which is characterized by dispersed hyphae, pellets, flocs, and other forms, is closely related to rheological properties, mass transfer efficiency, and metabolite production during fermentation. As a result, morphological control strategies have been developed from a basic process optimization to a comprehensive, multi-level strategy that integrates process parameters, environmental conditions, and genetic engineering. To provide an overview of the available intervention levers, we summarize representative morphology-engineering strategies, target outcomes, and selected case studies in Table 1, and discuss them in detail in the following sections.

**Table 1.** Summary of intervention strategies, target products, and representative cases in fungal morphology engineering.

Class	Intervention (set-point/manipulation)	Target product or metric (direction)	Strain/system	Reference
Inoculation & spore density (SmF/SSF)	SSF inoculum loading; optimum reported: 1 mL at 3.2 × 10^6^ spores.	Acidic protease (↑)	*Aspergillus* sp. Z1BL1	Usman et al. (2021)
	Inoculation mode: spore suspension vs. preculture (co-culture).	Butyrolactone profile (shift)	*A. terreus* (co-cultured with *S. rimosus*)	Boruta et al. (2024)
Culture environment (chemical cues)	pH set-point: pH 6.0 vs pH 5.0.	Hyperbranching phenotype (gated)	*A. niger* Δ*kexB*	van Leeuwe et al. (2020)
	pH-controlled fermentation (vs. uncontrolled pH).	Melanin (↑)	*Aur. melanogenum*	Zhou et al. (2023)
	Differentiation induction: nutrient depletion or methionine supplementation.	Cephalosporin C (CPC) (↑)	*A. chrysogenum*	Nash and Huber (1971)
Hydrodynamics & oxygen transfer	Agitation tuned to an intermediate “optimal shear window”.	Glucoamylase (↑ at intermediate shear)	*A. niger*	Kunz and King (2022)
	Co-optimization of shear × *K*_*L*_*a*.	Bikaverin (↑)	*F. oxysporum*	dos Santos et al. (2022)
	Agitation set-point optimization; optimum reported: 200 r/min.	Tannase (↑)	*A. niger*	Darah et al. (2011)
	Agitation-aeration set-point optimization; optimum reported: 800 r/min + 0.75 vvm.	Fructosyltransferase (↑)	*A. oryzae*	Maiorano et al. (2020)
Microparticle-enhanced cultivation	Talc microparticles: 5000 mesh, 4 g/L, added within 24 h.	Yellow pigment (↑)	*Monascus* spp.	Huang et al. (2021)
	Silicate microparticle addition.	β-mannanase (↑)	*A. sojae*	Yatmaz et al. (2016)
Morphological reprogramming	Random mutagenesis; selected for lower broth viscosity.	Cellulase (↑)	*T. reesei*	Adsul et al. (2022)
	Δ*ags1* (evaluated under KNO_3_ condition).	Lipids (↑)	*A. oryzae*	Wannawilai et al. (2020)
	Δ*rseA.*	Enzyme secretion (↑)	*A. nidulans*	Ogawa et al. (2021)
	Δ*agsA*Δ*agsB*Δ*sph3*Δ*uge3.*	Mycoprotein (39.98%↑)	*A. niger*	Zhou et al. (2025)
	CHS4 expression knockdown.	Penicillin (↑)	*P. chrysogenum*	Liu et al. (2012)
	Δ*chsA.*	Citric acid (↑); malic acid (↓)	*A. niger*	Zhu et al. (2023)
	Genetic perturbation of small GTPase.	Cellulase (↑)	*T. reesei*	He et al. (2016)

SSF, solid-state fermentation; SmF, submerged fermentation. *K*_*L*_*a*, volumetric oxygen mass transfer coefficient; vvm, volume of air per volume of liquid per minute. Arrows (↑/↓) indicate increase/decrease relative to the comparator in the cited study; “shift” denotes a change in product spectrum/profile.

### 4.1. Designing the “initial conditions” from spores to pellets

In submerged fermentation, the final hyphal structures are strongly influenced by spore hydrophobicity, viability, density, and aggregation. The spore clusters of different sizes can develop into pellets with different sizes and densities, shaping broth rheology and production patterns (Müller et al. 2022). In shake-flask, co-cultures of *Aspergillus terreus* and *Streptomyces rimosus* showed that inoculation significantly affected both secondary metabolite profiles and pellet size. Spore suspensions enhanced butyrolactone production of *A. terreus*, while preculture-based two-stage fermentation formed much larger pellets and a distinct secondary metabolite profile (Boruta et al. 2024). Thus, inoculation strategies are important drivers of morphological differentiation and metabolite patterns, and the single inoculation mode (e.g. a single cultivation setup or only a one-stage fermentation process) may underestimate biosynthetic potential. Similarly, the size and concentration of the inoculum also play a critical role in fungal morphology, biomass accumulation, and productivity in solid-state fermentation. An excessively high inoculum can lead to rapid growth and nutrient depletion, thereby inhibiting product formation, while insufficient inoculum results in inadequate biomass and low yield. For instance, an inoculum size of 1 mL (3.2 × 10^6^ spores/mL) of the *Aspergillus* strain Z1BL1 was found to achieve the highest crude acidic protease activity (107.4 U/mL) (Usman et al. 2021), demonstrating a distinct optimal inoculum range.

### 4.2. Reshaping morphology and metabolic networks via environmental control

As one of the important environmental factors, pH plays a critical role in morphological control including cell wall biosynthesis, branching patterns, and it may consequently influence product formation via changes in gene expression and related regulatory pathways. In *A. niger*, a *kexB* deletion strain (Δ*kexB*) exhibits significant hyperbranching on solid medium at pH 6.0 but not at pH 5.0, suggesting that external pH can be used for either amplifying or suppressing the effect of mutations related to cell wall and morphology, thereby offering a means to “guide” hyphal morphology to meet the process requirements (van Leeuwe et al. 2020). Similarly, a pH-controlled strategy significantly enhanced sporulation and increased melanin production by 178.6% in the fermentation of *Aureobasidium melanogenum* compared to an uncontrolled pH process, implying that pH regulation not only facilitates morphological differentiation but also augments production of the target metabolites (Zhou et al. 2023).

### 4.3. Co-shaping morphology and productivity via shear and oxygen supply

Agitation speed and aeration rate significantly affect shear stress and the volumetric oxygen transfer coefficient *K*_*L*_*a*, thereby influencing hyphal morphology and bioproductivity of filamentous fungi. In *A. niger*, agitation speed notably impacted the morphological transition from filamentous to pelleted forms and the tannase yield. At an agitation speed of 200 r/min, tannase production reached 1.41 U/mL with a biomass concentration of 3.75 g/L, representing the optimum within the studied range. Further increasing agitation speed enhanced mixing and oxygen transfer but may have caused excessive shear damage, thereby reducing both biomass and tannase production (Darah et al. 2011). For fructosyltransferase production in *A. oryzae*, agitation exerted a more pronounced effect on cell growth. Under condition of 800 r/min and 0.75 vvm, fructosyltransferase activity and productivity were maximized (Maiorano et al. 2020). These findings suggest that moderate shear stress promotes can favor advantageous pellet morphology and bioproduct yield, whereas excessive shear stress may disrupt fungal structure and reduce bioproduct secretion.

### 4.4. Engineering hyphal architecture using exogenous particles

Microparticle-enhanced cultivation (MPEC), which entails the incorporation of inert or functional microparticles to actively influence hyphal aggregation and pellet structure, has emerged as a significant process strategy for regulating fungal morphology. The spatial distribution, size, and shape of microparticles are key determinants of the fungal hyphal volume fraction and pellet density. Notably, smaller particles are more effective in reducing pellet density (Dinius et al. 2024), which may improve nutrient and oxygen transfer and thereby increase bioproduct yield (Driouch et al. 2011). In the submerged fermentation of *Monascus* spp., the addition of microparticles significantly increased the yellow pigment production, which was accompanied by transcriptional upregulation of genes related to pigment biosynthesis (Huang et al. 2021). In *Aspergillus sojae*, incorporation of silicate microparticles was observed to alter pellet morphology, yielding pellets with diverse sizes or dispersed hyphae, and was associated with increasing β-mannanase production and a significant reduction in the by-product oxalic acid (Yatmaz et al. 2016). These results suggest that microparticles could enhance fermentation performance via physical alteration of morphology and potentially through physiological responses (Tong et al. 2022).

### 4.5. Morphological reprogramming via the cell wall and polarity axis

For improving performance of the industrial filamentous fungi, the components of cell wall and polar growth axis are pivotal targets for morphological modification. Through random mutagenesis, the morphology of *T. reesei* has been improved, in which cellulase production was elevated and broth viscosity was clearly reduced (Adsul et al. 2022), indicating that alterations in cellular morphology and rheological properties can be synergistically optimized to improve enzyme production.

Alteration of cell wall biosynthesis can affect hyphal morphology and may improve fermentation efficiency. In *A. oryzae*, deletion of the β-glucan synthase gene *ags1* stimulated the formation of loose and small pellets. When KNO_3_ was used as the nitrogen source, this strain exhibited a marked increase in lipid production compared to the wild type (Wannawilai et al. 2020). In *A. nidulans*, deletion of *rseA*, which encodes a putative GT2 glycosyltransferase, triggered a cell wall stress response and enhanced enzyme secretion (Ogawa et al. 2021). Furthermore, simultaneously deleting *agsA*, *agsB*, *sph3*, and *uge3*, which are involved in α-1,3-glucan biosynthesis of *A. niger*, significantly improved mycelial dispersion and increased mycoprotein production by 39.98% in the submerged culture (Zhou et al. 2025). These findings highlight the potential of engineering cell wall polysaccharides to enhance fermentation performance.

In *P. chrysogenum*, reducing expression of the class III chitin synthase gene *chs4* brought about the shorter and more highly branched hyphae, while penicillin production increased by 20%–40% (Liu et al. 2012). Similarly, deletion of the chitin synthase gene *chsA* resulted in formation of smaller, loosely packed pellets with diminished cell wall integrity and an altered acid profile, which was characterized by decreased levels of malic acid and certain enzymes, yet significantly elevated citric acid production (Zhu et al. 2023). In *T. reesei*, the hyphae were transferred from the long and sparsely branched forms to the short and highly branched structures through genetic engineering of the small GTPases and the regulators for polarity and branching. This modification significantly increased the number of hyphal tips and the effective secretory surface area and was associated with enhancing the secretion of extracellular enzymes (He et al. 2016).

Collectively, the approach for morphological control in filamentous fungi has progressed from basic process optimization to a comprehensive, multidimensional engineering framework. This framework integrates inoculation strategies, environmental parameters (such as temperature and pH), agitation and aeration techniques, microparticle addition, and genetic modification (Figure 3). Morphology is now considered not merely a passive result but an actively designable and more precisely controllable “engineering variable”.

**Figure 3.**
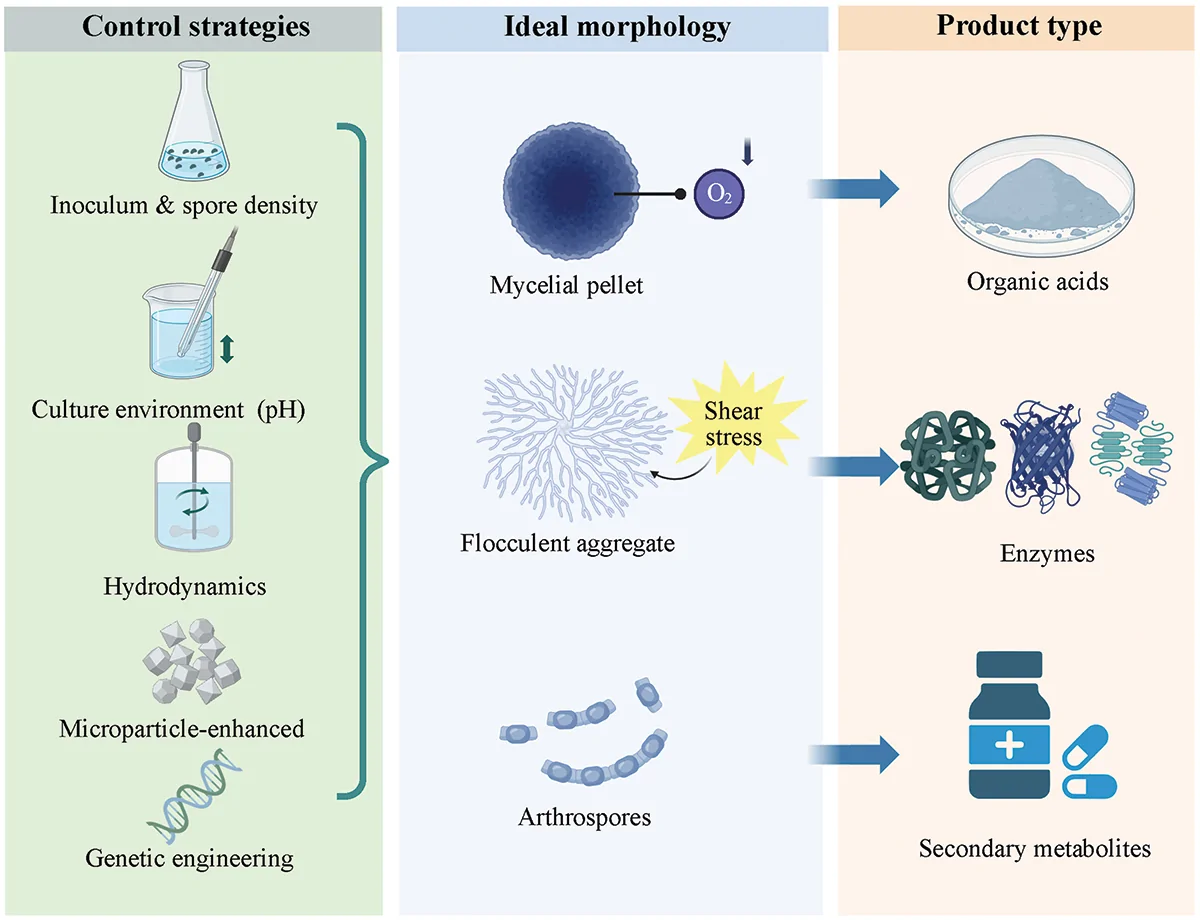
Morphology control strategies of filamentous fungi in industrial fermentation. Schematic overview of fermentation control strategies discussed in this review (inoculum/spore density, pH, agitation/aeration, microparticle-enhanced cultivation, and genetic engineering) and their links to desirable morphologies and typical product classes. Pelleted growth is often associated with organic-acid production and is influenced by internal O_2_ gradients and mass transfer. Dispersed mycelia/flocs are commonly favored for enzyme production, with shear shaping fragmentation/aggregation and secretion. Arthrospore-like morphologies may be relevant to secondary metabolite production in selected systems. Arrows denote conceptual associations rather than strict causal relationships.

## 5. Applications of morphology engineering in filamentous fungi

The morphogenesis of filamentous fungi could be manipulated through designing and engineering. By precisely adjusting morphology through process conditions, genetic modifications, and material interactions, productivity of the filamentous fungi can be enhanced in traditional industries such as biofuels (Hinterdobler et al. 2021), bioprocessing (Mäkelä et al. 2016), food and feed, and environmental remediation (Ghosh et al. 2023). Concurrently, a variety of emerging applications are broadening the potential of fungal morphology engineering, which include utilizing filamentous fungi as single-cell oil producers for biodiesel (Mhlongo et al. 2021), developing biodegradable biomaterials derived from the chitin-rich fungal cell wall (Zhang et al. 2022; Erdoğmuş et al. 2023), and employing fungal cell walls and hyphal networks as scaffold materials for tissue engineering and regeneration (Narayanan et al. 2020). All these applications necessitate a comprehensive understanding and precise control of morphogenesis, thereby imposing more stringent requirements on real-time, quantitative monitoring of morphological differentiation in filamentous fungi. Within this framework, advanced imaging techniques, mathematical modeling, and machine learning are increasingly recognized as essential methodological pillars propelling morphogenesis research towards becoming quantifiable, predictable, and designable (Table 2).

**Table 2.** Overview of emerging technologies facilitating the observation, quantification, and prediction framework in fungal morphology engineering.

Category	Methodology	Key function	Representative application
Advanced imaging	Stereomicroscopy with focus stacking (z-stacking)	Enables non-destructive 3D visualization of spatial organization in opaque matrices.	Spatial distribution in solid-state fermentation of *P. simplicissimum* (de Almeida et al. 2017)
	Time-lapse microscopy	Captures real-time growth dynamics and kinetic parameters from single cells to populations.	Single-spore development of *P. expansum* (Gougouli and Koutsoumanis 2012)
	X-ray micro-computed tomography (μCT)	Provides non-invasive 3D analysis of pellet internal microstructure, porosity, and microparticle distribution.	3D visualization of *A. niger* pellet microstructure (Schmideder et al. 2019, 2020; Deffur et al. 2024; Dinius et al. 2024)
Image analysis	MATLAB-based automated image analysis pipeline	Enables high-throughput quantification of culture heterogeneity, pellet size distributions, and fragmentation.	Discrimination of pellets vs. dispersed hyphae in *A. niger* submerged cultures (Müller et al. 2022)
	HyphaTracker (ImageJ-based)	Automates extraction of morphological descriptors (e.g. circularity, branching patterns).	Spore germination and hyphal network quantification in *Fusarium fujikuroi* (Brunk et al. 2018)
Modeling and simulation	Genome-scale metabolic models (GSMMs)	Integrates flux balance analysis with optimization (e.g. genetic algorithms) to predict multi-target constraints and prioritize experimental designs.	Optimization of organic acid production in *A. niger* (Upton et al. 2020)
	3D agent-based modeling (bmx/AMReX)	Simulates hyphal mechanics, nutrient transport, and network-topology evolution on adaptive meshes.	Morphogenesis in complex flow fields (Palmer et al. 2025)
Machine learning	Deep convolutional neural networks (CNNs)	Enables accurate automated recognition and classification of fungal colony morphologies.	Species identification and contamination detection in *Aspergillus* spp. (Ma et al. 2021)
Modeling and simulation	Multiphase artificial neural networks (MANNs) with NARX (exogenous inputs)	Predicts biomass dynamics in real time when direct measurements are impractical (e.g. insoluble substrates).	Biomass estimation of *Trichoderma* during cellulase production (Murugan and Natarajan 2019)
	Semi-supervised autoencoder + CNN	Predicts gene functions by integrating network topology and multi-omics features.	Identification of novel morphological regulators from multi-omics data (Peng et al. 2020)

NARX, nonlinear autoregressive model with exogenous inputs; ImageJ, open-source image analysis software.

### 5.1. Morphological observation and quantification

Previous investigations in the morphology of filamentous fungi have predominantly relied on two-dimensional microscopic images and empirical descriptions, which limit the ability to observe dynamic changes within complex three-dimensional structures over time. However, recent advancements in microscopic and tomographic imaging techniques have established a robust technical foundation for comprehensive morphological analysis, ranging from individual spores to extensive mycelial networks, and encompassing both solid-state and submerged fermentation processes.

In solid-state fermentation, correlations between fungal growth characteristics and production performance remain inadequately understood. Using a stereomicroscope with focus Z-stacking, researchers have successfully visualized the three-dimensional spatial distribution of *P. simplicissimum* without disrupting the matrix structure (de Almeida et al. 2017). This analysis of spatial organization offers critical insights into gas exchange, moisture distribution, and nutrient uptake within solid-state fermentation systems. In a study of *Penicillium expansum*, an autofocusing and extended depth-of-field (EDF) system alongside time-series imaging and frame-by-frame analysis was incorporated using time-lapse microscopy, which allowed for the direct acquisition of kinetic parameters including germination time and initial growth rate (Gougouli and Koutsoumanis 2012).

Recent advancements in image analysis techniques facilitate the rapid extraction of quantitative metrics from image data, supporting systematic development of growth-morphology models across strains (Jung 2018). In submerged fermentation, *A. niger* exhibits significant heterogeneity. By capturing time-series images over a 48 h period using a stereomicroscope and implementing an automated image analysis pipeline in MATLAB, spores, spore clusters, pellets, and dispersed hyphae were successfully differentiated. This approach enabled the quantitative assessment of their size distributions and area fractions, thus providing a measure of culture heterogeneity and the degree of pellet fragmentation (Müller et al. 2022). These image-analysis tools not only offer high-throughput morphological statistics but also enable the use of “morphology distributions” as indicators for process control and as inputs for modeling. Furthermore, the ImageJ-based HyphaTracker toolbox is capable of autonomously processing microscopic images to identify and extract regions pertinent to spore germination, and it has been used to compute parameters such as area, circularity, and time, thereby facilitating the efficient quantification of the germination process (Brunk et al. 2018).

Three-dimensional X-ray micro-computed tomography (μCT) and synchrotron radiation micro-CT (SR-μ-CT) have facilitated the non-destructive acquisition of comprehensive three-dimensional internal structures of fungal pellets. Utilizing μCT, pertinent studies have systematically examined the micro-morphology of intact fungal pellets, leading to the development of spatial models for nutrient and oxygen diffusion as well as mass transfer within the pellets (Schmideder et al. 2019, 2020; Deffur et al. 2024). In the context of microparticle-enhanced cultivation (MPEC) research, SR-μ-CT has been employed to conduct three-dimensional analyses on 375 *A. niger* pellets subjected to various MPEC conditions (Dinius et al. 2024). This analysis enabled the quantification of volume fractions of glass microparticles and fungal hyphae, and for the first time in three dimensions, accurately illustrated the spatial distribution pattern of exogenous microparticles within fungal pellets.

### 5.2. Modeling and machine learning for morphology–metabolism–process integration

The morphological differentiation of filamentous fungi represents a fundamentally multiscale nonlinear process characterized by cell wall formation, polarized growth, branching and fusion, network remodeling, and interaction with macroscopic flow fields. This complexity renders it challenging to be fully comprehended through empirical methods alone (Sarmah et al. 2022). The advent of mathematical modeling and machine learning offers novel methodologies for analyzing and predicting these intricate behaviors.

Fungal metabolic networks are large and highly redundant, so single-gene knockout often fails to yield the expected phenotype. Using an in silico evolution framework coupled with flux balance analysis (FBA), Upton et al. (2020) developed quantitative strategies to optimize *A. niger* fermentations for producing diverse organic acids with only limited experimental data. They integrated updated genome annotations and literature evidence to construct a genome-scale metabolic model (GSMM), iDU1756, and defined an approximate fitness function based on fermentation kinetics. By tuning flux bounds (to mimic knock-down/overexpression) and using genetic algorithms, they identified multi-target interventions for improving organic acid production, notably by limiting the electron transport chain, rerouting flux at the glyoxylate shunt and C3/TCA nodes, and boosting efflux capacity. This study illustrates the potential of employing “in silico evolution combined with flux-boundary optimization” as a preliminary step to wet-lab experiments, thereby expediting the narrowing of the engineering search space and generating system-level, testable hypotheses.

At a more granular spatial scale, a 3D modeling platform was developed utilizing the high-performance computing framework bmx/AMReX (Palmer et al. 2025). Employing verifiable metrics, this platform accurately replicates the morphological network observed in experimental settings, thereby offering a scalable simulation tool for the investigation of morphogenesis, nutrient transport, and the evolution of network topology of filamentous fungi in complex environments.

In the context of image classification, models based on deep convolutional neural networks (CNNs) have demonstrated the capability to classify images of *Aspergillus* colonies with remarkable accuracy, achieving rates as high as 99.7% (Ma et al. 2021). This advancement significantly enhances the efficiency of species identification and establishes a technical foundation for online morphology monitoring, contamination detection, and the assessment of strain stability. In the context of process monitoring, a dynamic soft sensor was developed based on a multiphase artificial neural network (MANN) to predict the biomass of *Trichoderma* during fed-batch fermentations involving insoluble substrates (Murugan and Natarajan 2019). This innovation provides a novel approach for achieving online estimation and control in systems where direct measurement of biomass is challenging, thereby enhancing cellulase productivity.

A significant number of these functions are intricately linked to morphology-associated processes, including cell wall synthesis, polarized growth, and stress responses. Integration of multiple network topologies with deep neural networks enables the prediction of gene functions with relatively high accuracy (Peng et al. 2020). Moreover, machine learning has emerged as a crucial tool for “mining morphological regulators from omics data”.

## 6. Challenges and future perspectives

Despite advancements in imaging and machine learning offering new opportunities for studying morphological differentiation of industrial filamentous fungi, there are still multiple challenges in achieving “predictive morphology engineering” from laboratory settings to industrial reactors.

One of the bottlenecks lies in data standardization and reproducibility. Most studies lack the unified standards for describing hyphal or pellet morphology using quantitative descriptors (e.g. compactness, roughness, dispersion, and branching frequency). Image-analysis varies substantially in imaging resolution, threshold-based segmentation methods, and statistical reporting criteria, also resulting in poor comparability across publications and limiting data reuse and integration. Beyond morphology descriptors and image-processing pipelines, standardization is also lacking in the reporting of process conditions that shape morphology and productivity. The key variables such as spore viability, the shear environment, dissolved-oxygen fluctuations, and viscosity-mass transfer coupling are often simplified in reporting or not recorded quantitatively, thereby constraining interpretable and predictive modeling of morphology–process–product relationships.

In parallel, most datasets are collected at a single scale (e.g. single spores, pellets, or reactor scale), resulting in insufficient multiscale data integration and weak quantitative bridging between different scales. A major challenge for comprehensive prediction is to integrate single-cell dynamics, 3D morphological structures, and macroscopic fluid mechanics into a cohesive framework.

Meanwhile, correlations among cell wall composition, branching patterns, and metabolic product profiles have been reported, yet generalizable morphology–metabolism–process principles remain difficult to distill, especially under the multi-stress conditions of industrial settings. Additionally, strong correlations have been reported among cell-wall composition, branching patterns, and metabolic product profiles. However, generalizable principles linking morphology, metabolism, and process conditions remain to be distilled.

Another critical limitation is transferability across strains, species, media, and reactor configurations. Morphology–product relationships are often condition-dependent and may not extrapolate across scales or hardware. This calls for modeling strategies that explicitly quantify uncertainty and improve out-of-distribution generalization, rather than optimizing performance on a single well-controlled dataset. Accordingly, it is urgently needed to move beyond descriptive correlations toward models that generalize across strains, scales, and reactor configurations while providing calibrated confidence.

In future, AI approaches are expected to progress from phenotype recognition to predictive modeling, and ultimately inverse design of morphology–process–product relationships. Moving beyond purely black-box predictions, incorporating biological and physical priors—such as constraints imposed by mass transfer, rheology, and established polarity/cell-wall regulatory networks—should improve interpretability and enhance reliability across conditions.

To generate outcomes that translate to industrial practice, the field will likely benefit from “closed-loop” workflows that combine high-throughput, standardized morphology measurements with rapid predictive models linking morphology, operating conditions, and productivity, together with data-efficient search strategies (e.g. active learning or Bayesian optimization) that recommend operating conditions or genetic modifications to test while respecting practical constraints such as oxygen availability, viscosity limits, and shear tolerance. Ultimately, integrating data, models, and experimentation in this way should enable not only forecasting morphological trajectories but also the inverse design of actionable interventions—such as inoculation strategies, cultivation environments, hydrodynamic regimes, microparticle additions, and targeted genetic modifications—to improve both productivity and processability.

Overall, studies on the morphological differentiation of industrial filamentous fungi are transitioning from an empirical, descriptive stage toward an engineering science paradigm that is quantifiable, predictable and designable. Through tightly integrating experiments, modeling, and intelligent algorithms, it should be possible to establish general design principles for morphology engineering, thereby providing robust support for green manufacturing and the development of novel biomaterials.
